# Proliferative Activity of Liver Growth Factor is Associated with an Improvement of Cigarette Smoke-Induced Emphysema in Mice

**DOI:** 10.1371/journal.pone.0112995

**Published:** 2014-11-17

**Authors:** Álvaro Girón-Martínez, Sandra Pérez-Rial, Raúl Terrón-Expósito, Juan José Díaz-Gil, Nicolás González-Mangado, Germán Peces-Barba

**Affiliations:** Respiratory Research Group, Instituto de Investigación Sanitaria - Fundación Jiménez Díaz - CIBERES, Universidad Autónoma de Madrid (IIS-FJD-CIBERES-UAM), Madrid, Spain; McGill University, Canada

## Abstract

Cigarette smoke (CS)-induced emphysema is a major component of chronic obstructive pulmonary disease (COPD). COPD treatment is based on the administration of bronchodilators and corticosteroids to control symptoms and exacerbations, however, to date, there are no effective therapies to reverse disease progression. Liver growth factor (LGF) is an albumin-bilirubin complex with mitogenic properties, whose therapeutic effects have previously been reported in a model of emphysema and several rodent models of human disease. To approach the therapeutic effect of LGF in a model of previously established emphysema, morphometric and lung function parameters, matrix metalloproteinase (MMP) activity and the expression of several markers, such as VEGF, PCNA, 3NT and Nrf2, were assessed in air-exposed and CS-exposed C57BL/6J male mice with and without intraperitoneal (i.p.) injection of LGF. CS-exposed mice presented a significant enlargement of alveolar spaces, higher alveolar internal area and loss of lung function that correlated with higher MMP activity, higher expression of 3NT and lower expression of VEGF. CS-exposed mice injected with LGF, showed an amelioration of emphysema and improved lung function, which correlated with lower MMP activity and 3NT expression and higher levels of VEGF, PCNA and Nrf2. Taken together, this study suggests that LGF administration ameliorates CS-induced emphysema, highlights the ability of LGF to promote alveolar cell proliferation and may be a promising strategy to revert COPD progression.

## Introduction

Chronic obstructive pulmonary disease (COPD) is a major disease affecting millions of people worldwide. One of the major risk factors for COPD is cigarette smoke (CS), since about 90% of COPD patients are cigarette-smokers [1] **.** The toxic compounds present in CS are partly responsible for the disruption of the alveolar and capillary network in the lung. Specifically, it has been reported that there are greater numbers of apoptotic alveolar epithelial and endothelial cells in lung tissues of COPD patient than in control patients, which in turn can lead to the development of emphysema [2]. Moreover, an increase in cell death mechanisms in the lungs correlates with decreased expression of vascular endothelial growth factor (VEGF) [3], which acts as a mitogenic factor promoting survival and differentiation of endothelial and alveolar epithelial cells [4], [5].

Chronic exposure to CS implies the absorption of a large amount of chemical compounds with important oxidative activity. In addition, inflammatory cells release reactive oxygen/nitrogen species, which also contribute to the sustained oxidative/nitrosative burden in the lungs [6]. In the context of the inflammatory milieu as it occurs in COPD, nitric oxide reacts with reactive nitrogen species resulting in the formation of 3-nitrotyrosine (3NT), which is considered a marker of nitrosative stress and therefore inflammation [7]. Nuclear factor (erythroid-derived 2)-like 2, also known as NFE2L2 or Nrf2, is a transcription factor that regulates the expression of a variety of genes involved in the antioxidant response. Interestingly, Nrf2-deficient mice have early-onset and more extensive CS-induced emphysema compared with wild-type littermates, thus suggesting that Nrf2 protects against the development of emphysema [8].

Currently, treatment of COPD is based on the administration of bronchodilators and corticosteroids, however there are no effective therapies directed towards the regeneration of lost alveoli in emphysema. Cell-cell interactions between alveolar epithelial cells and other cell types are mediated by releasing growth factors including hepatic, keratinocyte, epithelial or vascular endothelial growth factor [9]–[11]. The repair mechanisms to restore the normal airway architecture are inefficient in patients with COPD. Therefore, the use of growth factors as promoters of cell proliferation mechanisms and differentiation have emerged as a promising strategies to stimulate tissue repair under injury conditions [12]. Liver growth factor (LGF) is an albumin-bilirubin complex with mitogenic properties described for the first time in rat liver [13]. The antifibrotic and antioxidant properties of LGF, as well its regenerative effects, have been described in several rodent models such as injured liver [14], Parkinson's disease [15], [16], testis degeneration [17], hypertension and atherosclerosis [18], [19]. In addition, we have previously described the beneficial effects of LGF in a rat model of ClCd_2_ induced-lung fibrosis [20] and more recently in AKR/J mice with CS-induced emphysema [21] **.** In this study, our purpose was to deepen the understanding of the regenerative properties of LGF in C57BL/6J mice with CS-induced emphysema and clarify, by the analysis of several markers of lung damage and proliferation, the mechanisms by which LGF promotes the improvement of the emphysematous profile.

## Materials and Methods

### Animals

C57BL/6J male mice (Charles River Laboratories) 8 weeks old were housed in the Inhalation Core Facility at the IIS-Fundación Jiménez Díaz (n = 40). Protocols were approved by the local Ethical Animal Research Committee at IIS-Fundación Jiménez Díaz. In all cases, the legislation regarding animal treatment, protection and handling was followed (RD 53/2013).

### CS exposure

Mice were divided into air-exposed mice and CS-exposed mice. Animals were exposed to a mainstream CS of 4 research non-filtered cigarettes (3R4F, University of Kentucky, Lexington, KY; 11 mg TPM, 9.4 mg tar and 0.73 mg nicotine per cigarette), per day (5 minutes per cigarette with 10 minutes smoke free intervals, 5 days a week) during 6 months. An optimal smoke/air ratio of 1/6 was obtained. Mainstream CS was generated by an exposure system and was drawn into the chambers using a peristaltic pump (KD Scientific, Inc.) reaching concentrations of 200 mg TPM/m^3^ (Dust Track Model 8520, TSI Inc.). Non-smoking mice were exposed to room air. Body weights were assessed at the beginning of the experiment and at the third, fourth, fifth and sixth months of CS exposure.

### Liver growth factor (LGF) purification and administration

LGF was purified from rat serum following the procedure previously reported [22]. Briefly, the purification procedure consisted essentially of three chromatography steps, employing Sephadex G-75, DEAE-cellulose and hydroxyapatite. The absence of other growth factors and/or contaminants in the LGF preparations was also assessed according to standard criteria [13], [15], [22]–[24]. All LGF preparations showed a single band in sodium dodecyl sulphate polyacrylamide gel electrophoresis (SDS-PAGE. LGF preparations were lyophilized and stored at 4°C. Before use, LGF was dissolved in saline solution (NaCl 0.9%; Braun). The dose of LGF has been optimized in previous pilot experiments. Six months after CS exposure, when emphysema is once established, animals were treated with 4 *i.p*. injections (twice a week for 2 weeks) of 150 µl of a solution containing 1.7 µg LGF per mouse.

### Fluorescence molecular imaging *in vivo* (FMI)

Mice were anaesthetized during the imaging acquisition with a mixture of 2% isoflurane, 2 L/min oxygen using an Inhalation Anaesthesia System (Harvard Apparatus). Body hair was removed by shaving and subsequent application of depilatory cream. Each mouse was injected intravenously with 2 nmol of a protease activable fluorescent probe (MMPSense 680; PerkinElmer, Inc.) diluted in 150 µl of phosphate buffer. MMPSense 680 (excitation: 680 nm; emission: 700 nm) emits in the near-infrared when activated by MMP-2, -3, -9 and -13. 24 h after last tobacco exposition and fluorescence probe administration, *in vivo* images were acquired using an IVIS-Lumina Imaging System (Caliper Life Sciences, Inc.) as described previously [25].

### Lung morphometry

Lungs were fixed intratracheally with 4% formalin (Sigma-Aldrich, Co.) at a pressure of 25 cmH_2_O overnight. Then, formalin fixed tissues were paraffin embedded and cut into 5-µm-thick sections that were stained with hematoxylin and eosin (H&E) according to standard protocols. Enlargement of alveolar spaces was quantified by measurement of the mean chord length (L_m_) and alveolar internal area (AIA) using an image analysis software (LeicaQwin) specifically designed by Leica. After imaging processing, the software displayed the total number of alveolar spaces in the field and the Lm and AIA values for each one. Histological images were selected following random criteria and captured with a videocamera (Leica Microsystems) coupled to an optical microscope (Olympus BX40). Analyses of 18 representative images per mouse were performed in duplicate by two blinded observers.

### Lung function

Maximum inspiratory volume (V_max_) at a pressure of 30 mbar was the parameter used to evaluate lung function on each mouse. This parameter was registered using a ventilator/respirator device designed especially for small animals [26].

### Histological Analyses

The immunohistochemical staining was performed on paraffin-embedded lung 5-µm-thick sections. Proliferating cells were detected using an anti-PCNA antibody (Sigma-Aldrich).

### Western blot analysis

20 µg of total lung protein extract were denaturalized at 95°C during 5 minutes. Electrophoresis was performed in a 10% sodium dodecyl sulfate–polyacrylamide gel (SDS-PAGE) and transferred to a PVDF membrane. Prior to antibody incubation, membranes were blocked 1 h with 5% non-fat powdered milk in tris-buffered saline (TBS) in order to block non-specific interactions. After incubation at 4°C overnight with VEGFA, Nrf2, tubulin (Novus Biologicals), PCNA (Sigma-Aldrich, Co.) and 3NT (Santa Cruz Biotechnology, Inc.) primary antibodies, membranes were washed three times with TBS-0.5% Tween and incubated at room temperature during 1 h with secondary anti-rabbit antibody conjugated with horseradish peroxidase (Biolegend, Inc.). Finally, bands were visualized using a chemiluminescence detection kit (ECL Plus; GE Healthcare) according to the manufacturer’s instructions and quantified with Quantity-One Software (Bio-rad). Tubulin bands were used to normalize protein loading.

### Enzyme-Linked Immunosorbent Assay (ELISA)

Using total protein extracts from lung tissue homogenates, ELISA was performed with a mouse specific VEGF ELISA kit (ref: CSB-E04756m; CUSABIO), as indicated in the manufacturer’s protocol. The same amount of total protein was added on each well.

### Gelatin Zymography

Samples were electrophoresed onto a 10% polyacrylamide gel containing 1 mg/mL gelatin as substrate. On each electrophoretic lane, the same amount of total protein supernatants was electrophoresed. The gels were soaked with renaturating buffer (2.5% Triton X-100 in distillated water) at 37°C for 1 h to remove the SDS. After incubating the gels for 24 h at 37°C in the metalloproteinase buffer (50 mmol/L Tris-HCl, pH 7.4, 10 mmol/L CaCl2, 1% Triton-X100, 0.02% NaN3), they were stained for 30 min with 0.4% Coomassie blue to visualize bands of proteolytic activity and rapidly destained with 30% methanol and 10% acetic acid. The relative density of gelatinolytic bands was determined from scanned images of gels using image analysis software (ImageJ).

### RNA Isolation and Real-Time PCR

Total RNA was isolated using trizol reagent from frozen lung samples and reverse-transcribed to cDNA. For real-time PCR, we used TaqMan universal master mix and Taqman gene expression assays for the following genes: mmp9 (Mm00442991_m1), mmp2 (Mm00439498_m1), timp1 (Mm00441818_m1) and timp2 (Mm00441825_m1) (Applied Biosystems). 2-ΔΔCT method was applied to get the gene expression data using the Rn18s gene as an internal control to normalize the expression of the target genes mentioned above.

### Statistical analysis

Results were expressed as mean ± SEM. Statistical significance was taken as a p-value of less than 0.05 (*P*<0.05). Mann-Whitney method was performed to test significant differences between experimental groups followed by Monte Carlo's exact methods within each set of comparisons using the Statistical Package for the Social Science software (SPSS, Inc.).

## Results

### Body weight gain during CS exposure period

**Figure 1:**
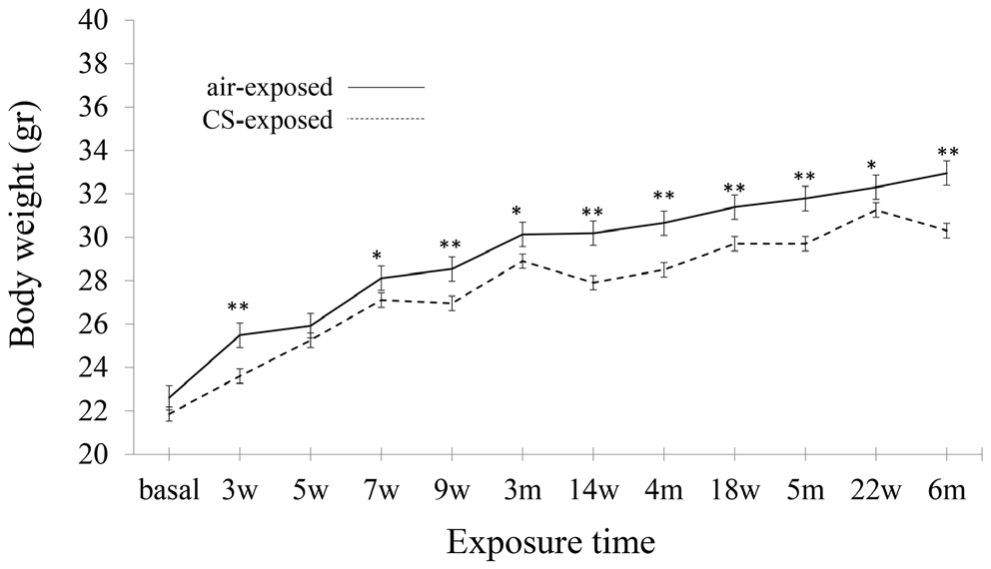
Body weight gain during CS exposure period. Solid line represents body weights of air-exposed group and dashed line represents body weights of CS-exposed mice (n = 40). * *P*<0.05 and ** *P*<0.01 *vs.* air-exposed mice. Data are presented as mean ± SEM.

Mice were exposed to CS or room air for 6 months. To evaluate the effect of CS inhalation, body weights of CS-exposed mice and air-exposed mice were assessed periodically. We observed that body weight gain in CS-exposed mice (28.9±0.31 gr) was significantly attenuated when compared to air-exposed mice (30.13±0.51 gr) at the third month of the study. Moreover, body weight gain was stabilized in the CS-exposed group until the end of the experiment, whereas in the air-exposed group the mice continued to gain weight (Fig. 1).

### Experimental emphysema is reversed morphologically and functionally by LGF

**Figure 2:**
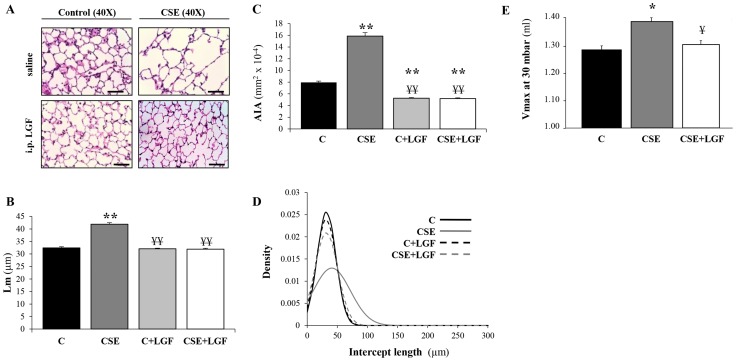
Alveolar space enlargement and lung function. (**A**) Histological sections from the lungs stained with H&E (n = 5 per group). Scale bars = 50 µm. Mean chord length (L_m_) (**B**) and the mean of the alveolar internal area (**C**) of alveoli in the lungs of air-exposed mice untreated (C) and treated with LGF (C+LGF) and CS-exposed mice untreated (CSE) and treated with LGF (CSE+LGF). (**D**) Distribution analysis of random intercepts obtained from measurements on randomly sampled images on linear scale (n = 5 per group). (**E**) V_max_ was used to evaluate lung function of air-exposed mice (C), CS-exposed mice (CSE) and CS-exposed mice post-treated with LGF (CSE+LGF) (n = 5 per group). * *P*<0.05 and ** *P*<0.01 *vs.* air-exposed mice; ¥ *P*<0.05 and ¥¥ *P*<0.01 *vs.* CS-exposed mice. Data are presented as mean ± SEM.

L_m_, AIA and V_max_ parameters were used to assess the degree of lung damage and development of emphysema caused by CS inhalation. When comparing representative images of each group, it was evident that the lungs from mice exposed to CS showed alveolar space enlargement. However, the morphology of alveolar spaces in lungs from mice exposed to CS and then treated with LGF was similar to that observed in normal lungs (Fig. 2A). As measured by mean chord length (L_m_) and alveolar internal area (AIA), air-exposed mice presented normal alveolar architecture (L_m_ = 32.46±0.46 µm; AIA = 794.5±23.39 µm^2^). In contrast, CS-exposed mice presented enlargement of alveolar spaces (L_m_ = 41.88±0.64 µm; AIA = 1587.19±59.54 µm^2^) (Fig. 2B–C). Lung changes observed in CS-exposed mice were substantially reversed in mice treated with LGF for two weeks after CS exposure (L_m_ = 32.04±0.35 µm; AIA = 517.18±13.13 µm^2^) (Fig. 2B–C). Additionally, analysis of intercepts lengths distribution in each group showed that in normal lungs, the density curve of the raw data had a slimmer and higher peak, whereas in lungs from mice with emphysema-like pathology (CS-exposed mice), the density curve had a wider but lower peak due to the disruption of alveolar septa (Fig. 2D). The shape of the density curve corresponding to the distribution of the intercept lengths in lungs from mice exposed to CS and then treated with LGF was more similar to that observed in normal lungs, suggesting that LGF was promoting tissue repair.

Regarding lung function, CS-exposed mice showed a consequent loss of lung function estimated by a significant increase in V_max_ (1.39±0.031 ml) when compared to the values in normal lungs (V_max_ = 1.29±0.058 ml). However, the administration of LGF significantly improved the status of lung function in mice exposed previously to CS (V_max_ = 1.30±0.044 ml) showing values of V_max_ similar to those seen in the control group (Fig. 2E).

It is important to note that air-exposed mice treated with LGF presented normal lung architecture compared with the untreated air-exposed group (Fig. 2A–D). Thus, we could verify that LGF treatment had no apparent negative consequences in the lungs of healthy animals.

### Lower MMP activity correlates with the reversion of the emphysema

**Figure 3:**
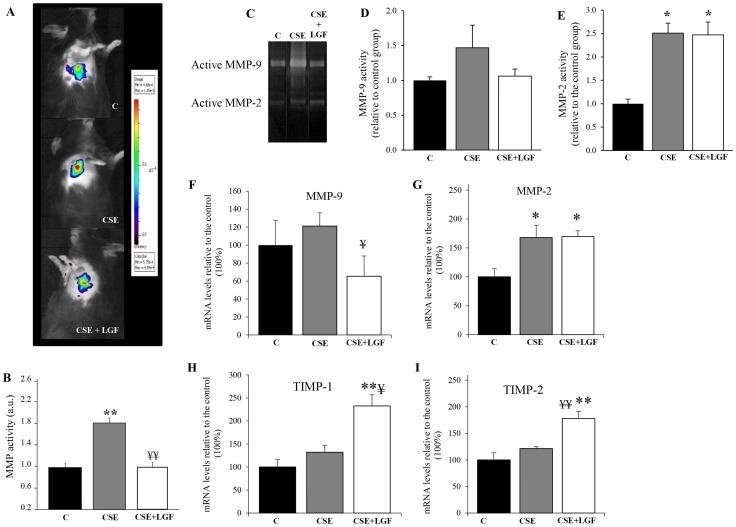
MMP activity. (**A**) In vivo MMP activity by FMI. Images were captured using the IVIS Imaging System. The entire efficiency signal from the thorax was measured with Living Image software using a spectral unmixing technique. One representative mouse of each group is shown. (**B**) Bars represent MMP activity in the lungs from air-exposed group (C), CS-exposed group (CSE) and LGF-treated CS-exposed group (CSE+LGF). The units used for the analysis of images were the ratio between emission and excitation light (referred to as efficiency). (**C**) Representative gelatin zymography indicating the activities of MMP-9 (**D**) and MMP-2 (**E**) in normal lungs and in lungs tissues from mice expose to CS and then non-treated or treated with LGF. Administration of LGF attenuated MMP-9 activity in CS-exposed mice. However, the MMP-2 activity was not attenuated by LGF administration. The expression of MMP-9 (**F**), MMP-2 (**G**), TIMP-1 (**H**) and TIMP-2 (**I**) mRNA relative to 18S rRNA were analyzed by real-time PCR. Results were expressed in % relative to non-exposed and non-treated mice that were arbitrarily assigned a value of 100%. * *P*<0.05 and ** *P*<0.01 *vs.* air-exposed mice; ¥ *P*<0.05 and ¥¥ *P*<0.01 vs. CS-exposed mice (n = 5 per group). Data are presented as mean ± SEM.

MMP activation promotes the destruction of extracellular matrix leading to an enlargement of alveolar spaces. Under normal conditions, tissue inhibitors of metalloproteinases can regulate MMP activity, but this balance can be impaired under tissue damage situations. In our model of experimental emphysema, CS-exposed mice presented a 2-fold increase in MMP activity assessed *in vivo* by FMI (Fig. 3A–B). After treatment with LGF, there was a significant decrease in MMP activity in CS-exposed mice when compared to CS-exposed non-treated mice (Fig. 3A–B), reaching values similar to those seen in animals exposed to room air.

In order to evaluate the activation of MMPs individually, we performed a gelatin zymography assay (Fig. 3C). The results showed that increased MMP-9 activity was regulated by LGF when it was administered to CS-exposed mice, reaching values similar to those showed in control group, although the variations were not significant (Fig. 3D). However, LGF administration had no effect on MMP-2 activity (Fig. 3E). In relation to mRNA expression levels of MMP-9 and MMP-2 and its inhibitors TIMP-1 and TIMP-2, similar results were obtained (Fig. 3F–I) to those observed by gelatin zymography.

### Tissue levels of VEGF and PCNA

**Figure 4:**
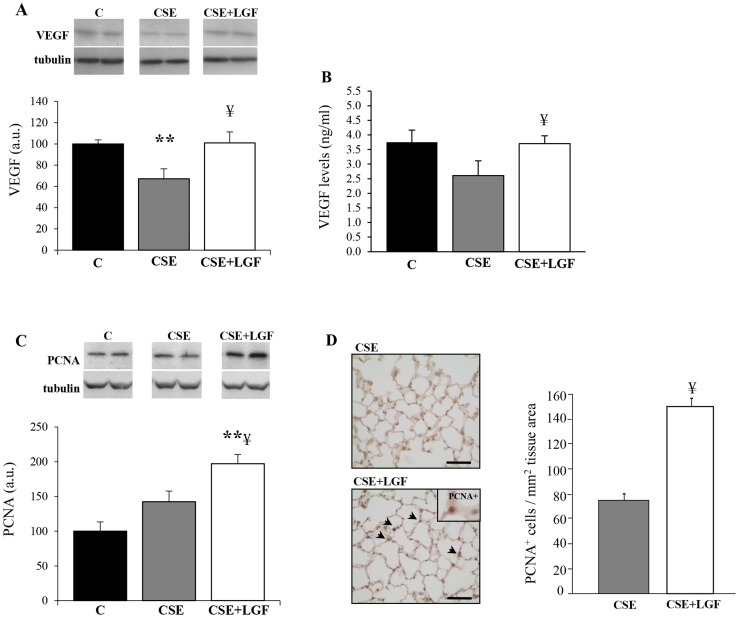
Tissue levels of VEGF and PCNA. VEGF levels were estimated by (**A**) western blot and (**B**) ELISA. (**C**) PCNA levels estimated by western blot. Data were normalized with tubulin. (**D**) Representative images of PCNA immunohistochemistry staining in the nucleus is shown for CS-exposed mice (CSE) and CS-exposed and LGF-treated mice (CSE+LGF). Scale bars = 50 µm. LGF promoted an increase of PCNA+ cells as showed in the bar graph. ** *P*<0.01 *vs.* air-exposed mice; ¥ *P*<0.05 *vs.* CS-exposed mice (n = 5 per group). Data are presented as mean ± SEM.

In order to assess the effects of LGF administration on cell proliferation, VEGF and proliferating cell nuclear antigen (PCNA) levels were determined in lung tissue homogenates. VEGF levels determined by western blot were diminished ∼30% in CS-exposed mice compared to the control group. But when treated with LGF, the amount of VEGF in lung tissue was similar than that observed in control group (Fig. 4A). In the same way, VEGF levels determined by ELISA were diminished in CS-exposed mice (2.6±0.8 ng/ml) and restored after treatment with LGF (3.48±0.7 ng/ml) (Fig. 4B). Regarding the amount of PCNA, there was an increase of ∼40% in CS-exposed mice treated with LGF compared to CS-exposed non-treated mice (Fig. 4C), which can be considered an index of the regeneration wave produced by LGF. Furthermore, a higher number of proliferating cells (PCNA^+^) was determined in CS-exposed and LGF-treated mice (151.3 cells/mm^2^) when compared to CS-exposed mice without LGF treatment (75.8 cells/mm^2^) (Fig. 4D).

### LGF ameliorates oxidative stress

**Figure 5:**
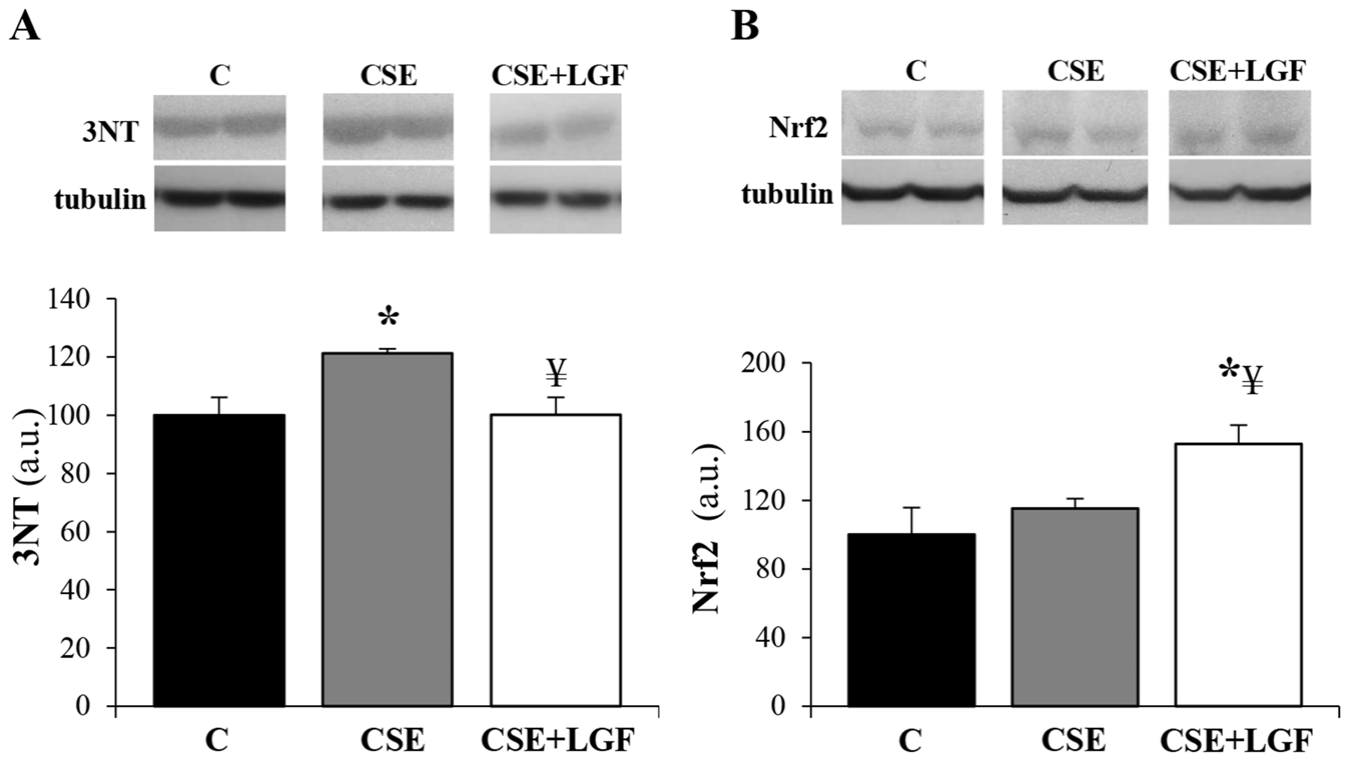
LGF ameliorates oxidative stress. Bars represent (**A**) 3NT and (**B**) Nrf2 levels estimated by western blot. Data were normalized with tubulin. * *P*<0.05 *vs*. air-exposed mice; ¥ *P*<0.05 *vs*. CS-exposed mice (n = 5 per group). Data are presented as mean ± SEM.

To study the effects of LGF administration on oxidative stress burden, 3NT levels (marker of oxidative stress) and NF-E2-related factor 2 (Nrf2) levels (marker of antioxidant response activation) were determined in lung tissue homogenates. Long-term exposure to CS induced a significant increase in 3NT (∼20%) compared to the control group. However, when CS-exposed mice were treated with LGF, 3NT expression reached similar levels to those seen in the control group (Fig. 5A). Moreover, Nrf2 expression did not present variations in CS-exposed mice in relation to control littermates, but increased ∼30% when treated with LGF (Fig. 5B).

## Discussion

CS-exposed mice developed lung emphysema as determined by the enlargement of alveolar spaces and loss of lung function, whereas the size of alveolar spaces in LGF-treated mice after CS exposure was similar to that seen in control mice. Moreover, lung changes correlated with an increase in MMP activity and 3NT expression and lower expression of VEGF in CS-exposed mice, while CS-exposed mice treated with LGF showed decreased MMP activity and 3NT expression, and higher levels of VEGF, PCNA and Nrf2. Thus, our study confirms that LGF treatment substantially reverses CS-induced emphysema as recently reported in AKR/J mice with CS-induced emphysema [21] and provides more details about the mechanisms that are regulated by the administration of LGF.

In a study performed in C57BL/6J mice [27], body weight gain was more attenuated in CS-exposed animals compared to the control group. Specifically, we confirmed that the body weight gain in CS-exposed mice was altered from the third week of CS-exposure (more consistently from the third month), whereas mice exposed to room air did not show any change. Similar alterations were also observed in a study performed by our group in AKR/J mice [28], where mice exposed to CS stopped gaining weight in the third month, and remained stable until the end of the experiment.

Over the years, many studies have focused on the use of therapeutic agents to reverse lung tissue disruption. In one study, all-trans-retinoic acid reversed emphysematous lesions in rats instilled with elastase [29], although not in adult mice [30]. N-acetylcysteine, another agent previously tested by our group, prevented morphometric and ventilation distribution alterations in the lungs of rats exposed to CS [31]. More recently, the use of growth factors has emerged as a new approach for the treatment of COPD [10]. Keratinocyte growth factor ameliorated the enlargement of alveolar spaces in C57BL/6J mice with elastase-induced emphysema [32]. Hepatic growth factor expression also improved the emphysematous changes in rats expressing human hepatic growth factor gene [33]. In our study, we tested the effects of LGF in pre-established CS-induced emphysema in C57BL/6J mice and showed that LGF improved lung changes as demonstrated by a decrease in L_m_ and AIA compared to CS-exposed and non-treated animals, reaching values similar to those of air-exposed group.

Alveolar macrophages activated by the toxic compounds in CS, release MMPs that participate in extracellular matrix degradation and contribute to emphysema [34]. In fact, several studies have shown higher levels of MMPs in patients with emphysema [35], [36]. Consistent with previous studies, here we show that long-term exposure to CS in mice leads to an increase in MMP activity in the lungs. In contrast, LGF treatment after CS exposure promoted a decrease of MMP activity as seen by *in vivo* FMI. The use of imaging techniques to visualize MMP activation in the lungs in a mouse model of CS exposure was previously described by our group [21], [25]. This method allows MMP activity to be assessed in real time, thus serving as a useful tool for evaluating lung damage.

According with the in vivo FMI experiments, the results obtained by gelatin zymography revealed that individual MMP-9 and MMP-2 activities, which were slightly increased in CS-exposed mice, were positively regulated by LGF administration. Although differences between groups were not significant, maybe due to the low number of mice per group, a tendency can be observed. Furthermore, the results of mRNA expression determined by real-time PCR correlated with those seen by gelatine zymography suggesting that LGF could regulate the extracellular matrix destruction by promoting the upregulation of MMP inhibitors TIMP-1 and TIMP-2 and the downregulation of MMP-9 but not MMP-2, both involved in the development of emphysema.

A study in rats showed that blocking the VEGF receptor increased alveolar enlargement and alveolar septal cell apoptosis [37]. Similarly, lung-targeted ablation of the VEGF gene led to air space enlargement in mice [38]. In this sense, our results showed that the amount of VEGF in lung tissues of mice exposed to CS was reduced, but restored when mice were treated with LGF after long-term CS exposure. Furthermore, it has been demonstrated that LGF is able to stimulate VEGF in rat testis [39] and to promote endothelial cell proliferation in different systems [18], [40]. PCNA was also upregulated in CS-exposed mice treated with LGF, which is considered to be a marker of LGF activity. In fact, several studies that addressed the effects of LGF in other disease models have revealed higher levels of PCNA, thus highlighting the mitogenic properties of LGF [22], [40], [41]. It is important to note that in lungs from mice treated with LGF after CS-exposure, there was a higher number of PCNA-positive cells determined by specific PCNA-staining, which seem to correspond to type II pneumocytes in terms of morphology, size and appearance. Type II pneumocytes are considered the progenitor cells of the alveolar epithelium with capacity to differentiate into type I pneumocytes in response to epithelial injury [42]–[44]. In fact, there have been many studies that have highlighted the important role of type II pneumocytes in alveolar epithelium repair [43]–[51]. In that sense, the administration of LGF could be promoting lung tissue repair by triggering the proliferation of type II pneumocytes.

Oxidative stress triggered by reactive chemicals present in CS may contribute to the abnormalities observed in the lungs of COPD patients such as destruction of the alveolar walls and enlargement of air spaces. In that sense, we observed that in lung tissues of CS-exposed mice, the levels of 3NT were increased when compared to control tissues. Our results are consistent with those described in patients with COPD, where the number of 3NT-positive cells and levels of 3NT increase in COPD airways [52]. Similarly, it has been reported that levels of 3NT are increased in the lungs of mice exposed to CS and negatively correlate with lung function [53]. Thus, we conclude that the decrease of 3NT observed in LGF-treated mice after CS exposure could be indicative of less oxidative burden and reduction of the inflammatory response in the lungs. We also showed that the expression of Nrf2 was not increased in CS-exposed mice. Furthermore, in Nrf2^−/−^ mice subjected to CS long-term exposure, it has been reported that neutrophil elastase is elevated when compared to wild-type mice, underlying the role of Nrf2 not only in the regulation of oxidant/anti-oxidant balance, but also in the regulation of inflammation and protease/anti-protease balance [54]. Similarly, our results showed that the post-administration of LGF in CS-exposed mice induced an increase in the expression of Nrf2 that correlates with less MMP activity *in vivo* and 3NT in lung tissues. Thus, our results suggest that Nrf2 could be playing an important role against the development of CS-induced emphysema, highlighting the positive impact of LGF in the activation of the anti-oxidant response. In line with this reasoning, LGF is known as a free radical scavenger.

Despite this, the mechanism of LGF action is very complex, due to the wide variety of the functions affected by LGF. For instance, LGF is able to stimulate the growth of hepatocytes, endothelial, smooth muscle cells, astrocytes, microglia and stem cells among others [18], [22], [40], [55]. This mitogenic activity of LGF in the liver is mediated by a local, transitory and mild TNF-α stimulation produced by endothelial cells [56]. On the other hand, LGF has also been shown to have antifibrotic activity in the liver [14] and is also a potent free radical scavenger, with both *in vivo* and *in vitro* activity [57]. Lastly, LGF injection promotes overstimulation of a wide number of intermediaries such as sphingosine 1-phosphate, a fundamental compound in cell survival in emphysema [58]. Our results deepen the understanding of the LGF regenerative properties in C57BL/6J mice with CS-induced emphysema and provide evidence, after analysis of lung injury markers and cell proliferation, about the mechanisms by which the LGF promotes improvement of emphysematous profile. Thus, further experiments are necessary to dissect the pathways activated and regulated by the action of LGF and to elucidate which cell types promote tissue repair.

Finally, the development of effective therapies to slow COPD progression is critical. Thus, we suggest that LGF treatment may be a promising strategy to reverse the progression of COPD in the future.
